# Improved algorithms for quantifying the near symmetry of proteins: complete side chains analysis

**DOI:** 10.1186/s13321-019-0360-9

**Published:** 2019-06-06

**Authors:** Inbal Tuvi-Arad, Gil Alon

**Affiliations:** 1 Department of Natural Sciences, The Open University of Israel, 4353701 Raanana, Israel; 2 Department of Mathematics and Computer Science, The Open University of Israel, 4353701 Raanana, Israel

**Keywords:** Protein structure, Side chains, Symmetry, Chirality, RMSD, Hungarian algorithm, Permutations, Molecular descriptors

## Abstract

Symmetry of proteins, an important source of their elegant structure and unique functions, is not as perfect as it may seem. In the framework of continuous symmetry, in which symmetry is no longer a binary yes/no property, such imperfections can be quantified and used as a global descriptor of the three-dimensional structure. We present an improved algorithm for calculating the continuous symmetry measure for proteins that takes into account their complete set of atoms including all side chains. Our method takes advantage of the protein sequence and the division into peptides in order to improve the accuracy and efficiency of the calculation over previous methods. The Hungarian algorithm is applied to solve the assignment problem and find the permutation that defines the symmetry operation. Analysis of the symmetry of several sets of protein homomers, with various degrees of rotational symmetry is presented. The new methodology lays the foundations for accurate, efficient and reliable large scale symmetry analysis of protein structure and can be used as a collective variable that describes changes of the protein geometry along various processes, both at the backbone level and for the complete protein structure.

**Electronic supplementary material:**

The online version of this article (10.1186/s13321-019-0360-9) contains supplementary material, which is available to authorized users.

## Introduction

Symmetry offers several advantages for the evolution, oligomerization and function of proteins [1–3]. It has been shown that symmetry leads to a reduction of errors in the process of protein synthesis, especially when long peptide chains are involved [1, 4]. Symmetry tends to increase the effectiveness of allosteric regulation, e.g., by the Monod–Wyman–Changeux model of allostery (also referred to as the symmetry model) [5]. Synthesizing a symmetric structure requires less information for coding the protein, therefore may lead to faster processes [1]. Usually closed symmetric systems tend to have lower energy than asymmetric ones as the interactions between the subunits are maximized due to the symmetry. Consequently symmetry could make proteins more stable and minimize unwanted excessive aggregation [6]. Indeed, symmetry is a characteristic of many protein structures [7]. Searching the Research Collaboratory for Structural Bioinformatics Protein Data Bank (RCSB PDB) [8, 9] for symmetric structures reveals that within ca. 145,000 structures, around 40% are symmetric, ~ 30% have cyclic symmetry with one rotation axis, and the other 10% have higher symmetry (e.g., dihedral, octahedral, icosahedral, etc.). Moreover, out of ~ 55,000 homooligomers in the database, 96% are symmetric.

Nevertheless, research shows time and again that protein symmetry is far from being perfect. Such imperfections have been related to several factors, among which are the function of the protein, thermodynamic considerations, and experimental conditions [10–12]. Quantification of these imperfections is still in its very beginning. The pioneering work of Zabrodsky Peleg and Avnir [13] introduced the Continuous Symmetry Measure (CSM)—a structural descriptor that translates the full collection of geometrical parameters that define a molecular structure into a single number that measures the distance of that structure from its nearest symmetric counterpart with respect to a given cyclic point group *G*. The group *G* can be generated either by a proper or an improper rotation. The latter case allows a calculation of the Continuous Chirality Measure (CCM) [14], as an important feature of the general CSM framework. The task of computing the CSM requires the solution of an optimization problem, in which the parameters are both a permutation of the molecule’s atoms and a three dimensional direction vector [15]. For macromolecules, the huge number of possible permutations makes the calculation (and even approximation) of the CSM a nontrivial algorithmic problem.

The first step towards an efficient method for calculating the CSM was the work of Pinsky et al. [15] who developed an efficient method for finding the optimal direction vector, for any given permutation. They also introduced a division of the molecule’s atom into certain equivalence classes, based on the atoms’ type and the connectivity map of the molecule [16]. This division narrows down the realm of possibilities for the atom permutation, but the number of permutations remains too large to enumerate, except for very small molecules.

A partial solution to this problem was given in the work of Dryzun et al. [17] who were the first to introduce an approximate method for the calculation of the CSM. Starting with a reasonable guess of the direction vector, a sequence of iterative steps is performed where in each step a new atom permutation is calculated from the direction vector, and a new direction vector is calculated from that permutation, until convergence is reached. While the method of calculating the vector from the permutation (similar to the strategy of Pinsky et al. [15]) is both efficient and exact, as we recently proved [16], the method of calculating the permutation from the direction vector uses a greedy algorithm to find the permutation, which is relatively fast, yet generally provides only a crude approximation as compared with the exact algorithm. Nevertheless, the method provided the first practical tool for estimating the CSM of proteins, macromolecules and nanomaterials [17].

In a somewhat different direction, we have recently developed an algorithm for an exact calculation of the CSM [16], which is feasible for small to medium sized molecules. This algorithm takes into account all the information from the connectivity map of the molecule, and enumerates only the permutations that preserve the molecule’s bonding structure.

All of the methods described above are insufficient for estimating the symmetry of proteins. For the exact method [15, 16], the number of atoms, and consequently the number of permissible permutations are simply too large for the algorithms to complete the calculation in a reasonable time. The approximate method of Dryzun et al. [17] is also ineffective for this case, because of accumulated inaccuracies due to the approximate scheme and the use of the greedy algorithm. Furthermore, disregarding the peptide structure produces symmetric structures that mix atoms from different peptides and violate chemical rules. Nevertheless, assuming the permutation is dictated by the serial numbers, that is, each atom is permuted to an atom from another peptide with the same sequence number, provides a good estimation of the CSM of protein homomers. Recently, Bonjack-Shterengartz and Avnir applied this strategy and showed the effectiveness of using the CSM to describe the near symmetry of proteins [10, 11]. As will be shown here, this method limits the precision of the CSM calculation, especially when the number of peptides increases.

Other methods for the estimation of the protein symmetry are discussed in the literature [18–27]. These are generally based on quaternary structure alignment algorithms that involve the superposition of two peptides one over each other, while estimating their alignment by either root mean square deviation (RMSD) of matching α-carbons, or by a related scoring formula (e.g., the combinatorial extension (CE) score [28] or the template modeling (TM) score [18, 29]). However, these methods generally ignore the geometry of the side chains and therefore do not attempt to find the true permutation of the atoms.

In this paper we describe a new method for estimating the CSM for proteins. Our method relies on the properties of the protein sequence and the division into peptides, and presents algorithmic improvements as compared with previous methods. Specifically, our strategy introduces three improvements over the method of Dryzun et al. [17]: (a) We take into consideration the information of the protein sequence. This allows us to refine the division of atoms into equivalence groups, such that only atoms of the same identity, residue type and residue sequence number can be interchanged. (b) The greedy algorithm for calculating the permutation in the iterative step is replaced with the Hungarian algorithm, which is known to guarantee to yield an optimal solution of the related assignment problem [30, 31]. (c) We take into considerations the division of the molecule into peptides. Our algorithm distinguishes between the permutation of the peptides and the permutation of the atoms in the peptides, thus making sure that the resulting permutation will not mix atoms from different peptides. The Hungarian algorithm is applied at this stage as well, to find the optimal peptide permutation. These improvements lead to a tremendous increase in the accuracy and speed of the calculation, and turn the CSM into a robust methodology to describe protein structure, that can be used both for homomers as well as internal symmetry investigation of protein domains.

## Methodology

### Review of the CSM

Let us briefly review the fundamentals of the CSM methodology [13, 15, 16]. We consider a given molecule *A* of *N* atoms, and a point group symmetry *G* of order *n*, which can be of the type *C*_*n*_ or *S*_*n*_. Let ${\mathbf{Q}} = \left\{ {{\mathbf{Q}}_{k} :1 \le k \le N} \right\}$ be the set of coordinate vectors of the molecule’s atoms, and let ${\mathbf{Q}}_{0} = \frac{1}{N}\sum_{k = 1}^{N} {{\mathbf{Q}}_{k} }$ be its geometric center of mass. We are looking for a symmetry operation $T$, which generates a cyclic point group of type *G*. Note that *T* is a rotation (either proper or improper) by an angle of ${{360^{ \circ } } \mathord{\left/ {\vphantom {{360^{ \circ } } n}} \right. \kern-0pt} n}$. In both cases, $T$ is determined by a 3-dimensional direction vector, which we denote by $v_{sym}$. The continuous symmetry measure (CSM) is defined by $S\left( G \right) = 100 \cdot {{M(G)} \mathord{\left/ {\vphantom {{M(G)} {N(G)}}} \right. \kern-0pt} {N(G)}}$, where

1 $$M(G) = \hbox{min} \left[ {\sum\limits_{k = 1}^{N} {\left| {{\mathbf{Q}}_{k} - {\mathbf{P}}_{k} } \right|^{2} } } \right];\quad N(G) = \sum\limits_{k = 1}^{N} {\left| {{\mathbf{Q}}_{k} - {\mathbf{Q}}_{0} } \right|^{2} }$$

and the minimum is over all the symmetric (i.e. $T$-invariant) structures $\left\{ {{\mathbf{P}}_{k} :\,\,1 \le k \le N} \right\}$ and all possible direction vectors $v_{sym}$. Each symmetric structure $\left\{ {{\mathbf{P}}_{k} } \right\}$ induces a permutation $\pi$ on the set of atoms $\left\{ {1,2,\ldots,N} \right\}$, defined by the relation

2 $$T{\mathbf{P}}_{k} = {\mathbf{P}}_{\pi (k)} \;{\text{for}}\;1 \le k \le N$$

A symmetric structure $\left\{ {{\mathbf{P}}_{k} } \right\}$ which minimizes Eq. (1) is determined by $T$ and the permutation $\pi$, via the relation

3 $${\mathbf{P}}_{k} = \frac{1}{n}\sum\limits_{i = 1}^{n} {T^{ - i} {\mathbf{Q}}_{{\pi^{i} (k)}} }$$

Therefore, the calculation of the CSM and the nearest symmetric structure amount to finding the vector $v_{sym}$ and the permutation $\pi$ which minimize Eq. (1), or equivalently, attain the minimum

4 $$M(G) = \frac{1}{2n}\hbox{min} \sum\limits_{i = 1}^{n} {\sum\limits_{k = 1}^{N} {\left| {T^{i} {\mathbf{Q}}_{k} - {\mathbf{Q}}_{{\pi^{i} (k)}} } \right|^{2} } }$$

The permutation $\pi$ must satisfy a few requirements:

- Since *T* is a generator of the group *G*, and $\pi$ is related to *T* by Eq. (2), the cycles of $\pi$ can only be of size 1, 2, or *n* (2 is only allowed when *G *= *S*_*n*_ or *G *= *C*_2_).
- $\pi$ must preserve the structure of the original molecule. This means that $\pi$ only permutes atoms of the same type, and that $\pi$ neither breaks the bonds between atoms, nor creates new ones.

A method for an efficient enumeration of the permutations satisfying these conditions was described in our previous publication [16]. However, for proteins, such an enumeration is impossible as there are just too many such permutations. In earlier implementations of the CSM calculation [15, 17], the structure preserving condition was not enforced, but rather a weaker condition was used: The molecule’s atoms were divided into equivalence classes $\left\{ {{\mathcal{C}}_{1} ,{\mathcal{C}}_{2} , \ldots } \right\}$. The division was determined by an iterative process in which the initial division is deduced from the atom types, and further refinements were based on neighboring atoms in the connectivity map of the molecule. Finally, the permutation was required to preserve the equivalence classes.

### Estimating the CSM for large structures

The approximate algorithm of Dryzun et al. [17] is based on an iterative calculation. One begins with a reasonable guess of the direction vector $v_{sym}$; and then, at each iterative step, a permutation is calculated from the current vector, and a new vector is calculated from that permutation. This is repeated until either the process converges (the value of $v_{sym}$ stabilizes) or too many iterations have passed. The initial guess of the direction vector is found by performing linear regression on the set of centers of mass of the equivalence classes $\left\{ {{\mathcal{C}}_{1} ,{\mathcal{C}}_{2} , \ldots } \right\}$. This is reasonable because for perfectly symmetric molecules, the symmetry operation preserve these centers of mass. The permutation is calculated in each iterative step by a greedy algorithm: In each step, an atom *i* and a permutation value $\pi (i) = j$ are chosen, such that: (a) $j$ is in the same equivalence class as *i*. (b) The atom *i* has not yet been assigned a permutation value and the atom *j* has not yet been assigned as a permutation value. (c) The distance $|T{\mathbf{Q}}_{i} - {\mathbf{Q}}_{j} |$ is minimal among such pairs *i,j*. The direction vector $v_{sym}$ is calculated from the permutation $\pi$ by observing that given $\pi$, the solution of Eq. (4) is a quadratic optimization problem, which can be efficiently and accurately solved using Lagrange multipliers and matrix eigenvalues [15].

The above algorithm for finding the permutation has the advantage of a reasonable running time, which makes it feasible for large molecules. However, it is not always accurate since the greedy algorithm does not take into account the interaction between the choices of permutation values for various atoms. An example of the inaccuracy created by the greedy algorithm even in the case of two atoms is given as Additional file 1.

#### Finding the permutation with the Hungarian algorithm

Our first improvement of the approximate method, which is not specific for proteins, focuses on the calculation of the permutation in the iterative step. We are given a direction vector $v_{sym}$ (determined in the previous step), and a symmetry operation *T*. For each equivalence class ${\mathcal{C}}_{i}$, consisting of the atoms $\left\{ {a_{1} , \ldots ,a_{k} } \right\}$ we define a $k$ × $k$ matrix *A*, whose elements, *A*_*ij*_, are given by:

5 $$A_{ij} = \left| {T{\mathbf{Q}}_{{a_{i} }} - {\mathbf{Q}}_{{a_{j} }} } \right|^{2} \quad {\text{for}}\quad 1 \le i \le k,\quad 1 \le j \le k$$

Our algorithm chooses the permutation $\pi$ of the values $\left\{ {a_{1} , \ldots ,a_{k} } \right\}$, defined by $\pi (a_{i} ) = a_{\mu (i)}$ (for $i = 1, \ldots ,k$), where $\mu$ is the permutation of $\left\{ {1, \ldots ,k} \right\}$ for which the sum $\sum_{i = 1}^{k} {A_{i\mu (i)} }$ is minimal. Therefore, $\mu$ is the solution of *the assignment problem* for the matrix *A* [30]. For this problem, there is an efficient algorithm—the so-called Hungarian Algorithm, which finds the optimal solution in time $O(k^{3} )$ [30]. We provide further information about the assignment problem and its well-known solution in the Additional file 1.

Our revised method consists of forming the matrix *A* for each equivalence class, and obtaining the permutation values in each equivalence class by running the Hungarian algorithm on this matrix.

It should be noted that our algorithm does not take into account the restriction on the cycle structure of the permutation, described above. To the best of our knowledge, there is no efficient solution to the assignment problem under such cycle structure constraints. We also note that our method for finding the permutation (like the method of Pinsky et al. [32]) aims to minimize the term in Eq. (4) corresponding to $i = 1$. This has proved to be a good approximation of the minimizer of the entire sum in Eq. (4).

### Reducing equivalence groups: the “use sequence” algorithm

It is intuitively clear, and practically verifiable, that a crucial factor in the accuracy and efficiency of the approximate algorithm (with the Hungarian method improvement) is the size of the atom equivalence classes: The algorithm has better performance when the classes are small. For proteins, we can greatly refine the initial division into classes by using the information of the protein sequence. We assign different classes to pairs of atoms which differ in their chemical identity, residue type or sequence number (excluding the remoteness indicator). Consequently the size of the equivalence classes is determined by either the number of peptides (for protein backbone atoms and most of the side chains atoms as well), twice this number for atoms that differ only by their remoteness indicator (e.g., the two C_γ_ atoms of Val), or three times this number if hydrogen atoms are taken into account.

### The many chains algorithm

We now describe a further improvement of the algorithm, in which we make sure that the permutation does not break the peptides, but rather, carry each peptide in its entirety to another peptide. This is achieved by performing two levels of implementation of the Hungarian algorithm. The lower level calculates the mapping of the atoms in a peptide, and the higher level calculates the permutation of the peptides.

**Fig. 1:**
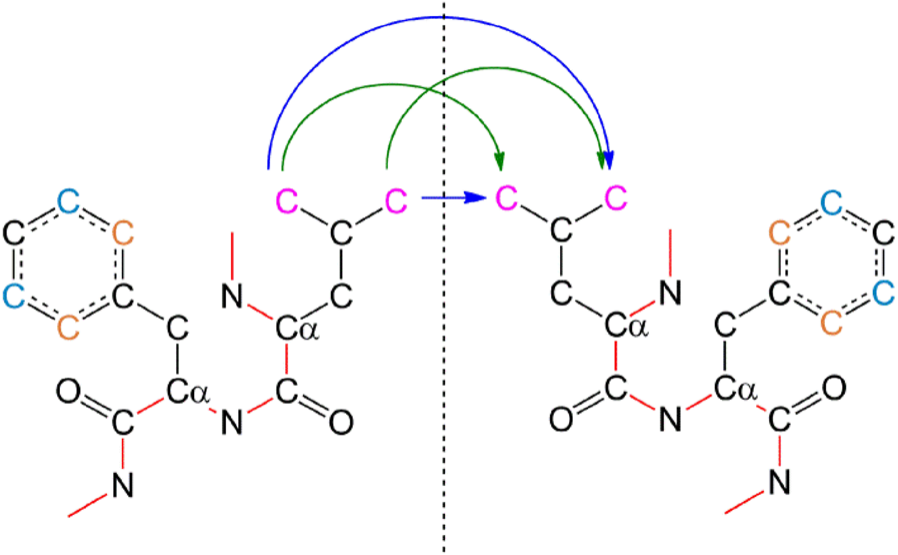
Atoms’ permutation for a two-residue (Leu-Phe) section of a homodimer. Black atoms form equivalence classes of two atoms each (one from each peptide). Orange, blue and magenta carbon atoms represent carbon atoms that differ only by their remoteness indicator, each couple forms an equivalence group of four atoms. Red bonds represent the protein backbone. Two choices for permuting the Leu-C_δ_ carbon atoms (magenta) are illustrated with blue and green arrows

Let us denote the peptides by $\left\{ {\mathsf{P}_{1} , \ldots ,\mathsf{P}_{\ell } } \right\}$. In the iterative step of the approximate algorithm, given the symmetry operation $T$, we calculate for each pair of peptides $\mathsf{P}_{i} ,\mathsf{P}_{j}$, the following minimum:

6 $$B_{i,j} = \hbox{min} \sum\limits_{u = 1}^{M} {\left| {TU_{i} - V_{\nu (i)} } \right|^{2} }$$

where $\left\{ {U_{1} , \ldots ,U_{M} } \right\}$ are the atoms of $\mathsf{P}_{i}$, $\left\{ {V_{1} , \ldots ,V_{M} } \right\}$ are the atoms of $\mathsf{P}_{j}$, and the minimum is over all the permutations $\nu$ of $\left\{ {1, \ldots ,M} \right\}$ which preserve the equivalence classes. The value of $B_{i,j}$ and the minimizing permutation $\nu$ is calculated by the Hungarian algorithm as explained above. We call this stage the lower level application of the Hungarian algorithm—see Fig. 1.

**Fig. 2:**
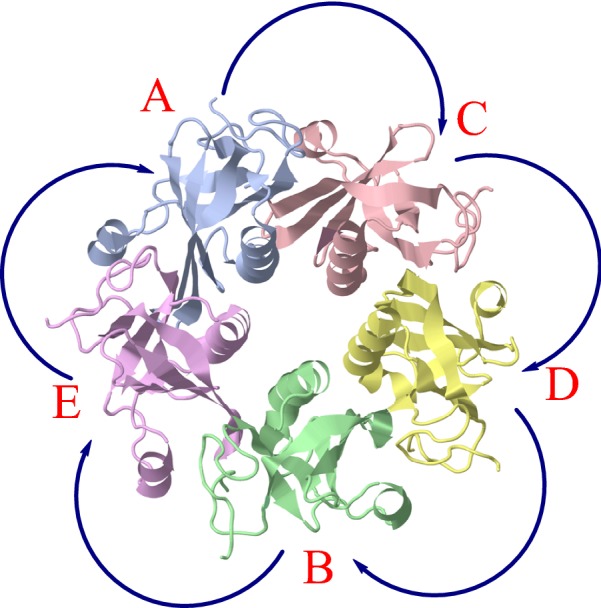
Peptides’ permutation in AB_5_ toxin from *E. coli* (PDB ID: 3DWA) fits the peptide permutation order: A → C → D → B → E. *S*(*C*_5_) = 0.0410. Peptides’ labels are taken from the PDB file

Applying the Hungarian algorithm to the matrix $B = \left( {B_{i,j} } \right)$ (this is the higher level application of the Hungarian algorithm, see Fig. 2) results with the optimal permutation of the peptides. Given the permutation of the peptides, and using the permutation calculated in the lower level, we obtain the full permutation of the molecule’s atoms. This permutation has the desired property of maintaining the peptides structure as well as the protein sequence, and is the optimal one among all such permutations.

### Testing methodology

#### Creating a database of the proteins: selection criteria

To test the effectiveness of our code we applied it to several sets of protein homomers. The coordinates of the proteins of each set were extracted from the RCSB website [8, 9]. In order to assure that only high quality proteins with minimal statistical bias will be selected [33, 34], several criteria for filtering the proteins were applied: (a) The experimental method was X-ray crystallography with a resolution of 2.5 Å or better, equivalent to at least a “Good” grade as defined by FirstGlance in Jmol [35]; (b) Only homomeric proteins were chosen in which the asymmetric unit contained all chains of the protein required to create a symmetric structure, that is, a biological assembly identical to the asymmetric unit exist, as defined by the transformation matrix in remark 350 of the PDB file. (c) DNA, RNA or hybrid chains were filtered out. (d) Based on R_free_ values the proteins were assigned with an R_free_ grade [36], which measures the quality of fitting a simulated diffraction pattern to the analyzed experimental diffraction pattern. Only proteins with a grade of “average at this resolution” and better were included; (e) finally, to reduce redundancy, proteins were filtered to maintain up to 70% sequence identity as defined by the RCSB website. Lists of the proteins used in this work are provided in the Additional file 1. Filtering was based on the RSCB search terms and followed by our own Python code: pdb_prep (see below). It should be noted that averaged B-factors, representing the mean square isotropic displacement of each atom [37, 38], were not required as an additional filter since for most of the protein used, the above filters naturally reduced these values to less than or equal to 40 Å^2^. However, those with higher average B factor showed good R_free_ grades and were therefore included in this study.

The above filters were applied to all symmetric homomers found in the RCSB website with *C*_4_, *C*_5_ and *C*_6_ rotational symmetry resulting with sets of 31, 51 and 16 proteins respectively. For protein homodimers with *C*_2_ symmetry and homotrimers with *C*_3_ symmetry, which are common in the RCSB database, we applied a randomization algorithm (by the Linux “shuf” command written by Paul Eggert) to choose 300 proteins of each type from the website. After applying the above filters these sets were reduced to 194 and 214 proteins respectively.

#### Preparing proteins for CSM calculations

Prior to analysis, each protein in our sets was cleaned with our python code pdb_prep to delete ligands, solvents, non-coordinates lines (e.g., ANISOU data representing anisotropic temperature factors) from the ATOM section in the PDB file [8, 9], and choose the first location in cases of alternate locations of specific residues. Hydrogen atoms, if existed, were deleted in order to unify the dataset, as most of the PDB files did not include them. In addition the code used reported data on missing residues and atoms (based on remarks 465 and 470 in the PDB file) to insure that all peptides have the same length. If a residue was missing from one or more of the peptides—it was automatically deleted from all other peptides. Similar treatment was given to missing atoms. Finally the files were checked to verify that the length of all peptides is identical.

## Results

### Symmetry levels of homomers

Table 1 presents descriptive statistics of our set of proteins. It should be noted that while the general scale of the CSM is between 0 and 100, typical CSM values of proteins with approximate symmetry are significantly smaller than 100 [10]. For our sets of proteins the distortion levels varied between 0 and 2. This does not necessarily mean that proteins are more symmetric than small molecules. Rather this results from the definition of the symmetry measure. The denominator, $\,N(G)$, in Eq. (1) is the sum of distances of each atom from the center of mass of the molecule. While for small molecules this value may be at the order of the sum of deviations of each atom from its expected symmetric position (as appears in the numerator, $M(G)$), for a protein, especially for an elongated structure, the sum of these distances can be much higher than the numerator leading to a small CSM value. Table 1 should thus be used as a reference table to which CSM levels of proteins with approximate symmetry can be compared. Within this range, up to 4 orders of magnitude differences exist between the minimum CSM representing highly symmetric proteins and the maximum CSM representing highly distorted ones. It should be noted that for asymmetric proteins, the CSM can be significantly higher than the values in Table 1, and values in the range 20–30 and even higher are common. On the other hand, there are specific structures that appear to be symmetric by their CSM value, although they are classified as asymmetric in the RCSB website. We comment on this topic in the Additional file 1 and presents statistics for asymmetric homotrimers and homotetramers in Additional file 1: Table S1.

**Table 1:** Descriptive statistics of CSMs for the sets of homomers

Set	CSM	N	Mean	Standard deviation	SE of mean	Minimum	Median	Maximum
Dimers	*S*(*C*_2_)	194	0.1350	0.3132	0.0225	0.0001	0.0471	2.8786
Trimers	*S*(*C*_3_)	214	0.0775	0.1698	0.0116	0.0003	0.0304	1.9519
Tetramers	*S*(*C*_2_)	31	0.0446	0.1444	0.0259	0.0006	0.0130	0.8171
	*S*(*C*_4_)	31	0.1001	0.2490	0.0447	0.0022	0.0311	1.0366
Pentamers	*S*(*C*_5_)	51	0.0974	0.1872	0.0262	0.0028	0.0345	0.9505
Hexamers	S(C_2_)	16	0.0529	0.0437	0.0109	0.0055	0.0435	0.1565
	S(C_3_)	16	0.0714	0.0603	0.0151	0.0077	0.0555	0.2326
	S(C6)	16	0.0898	0.0715	0.0179	0.0102	0.0737	0.2699

**Fig. 3:**
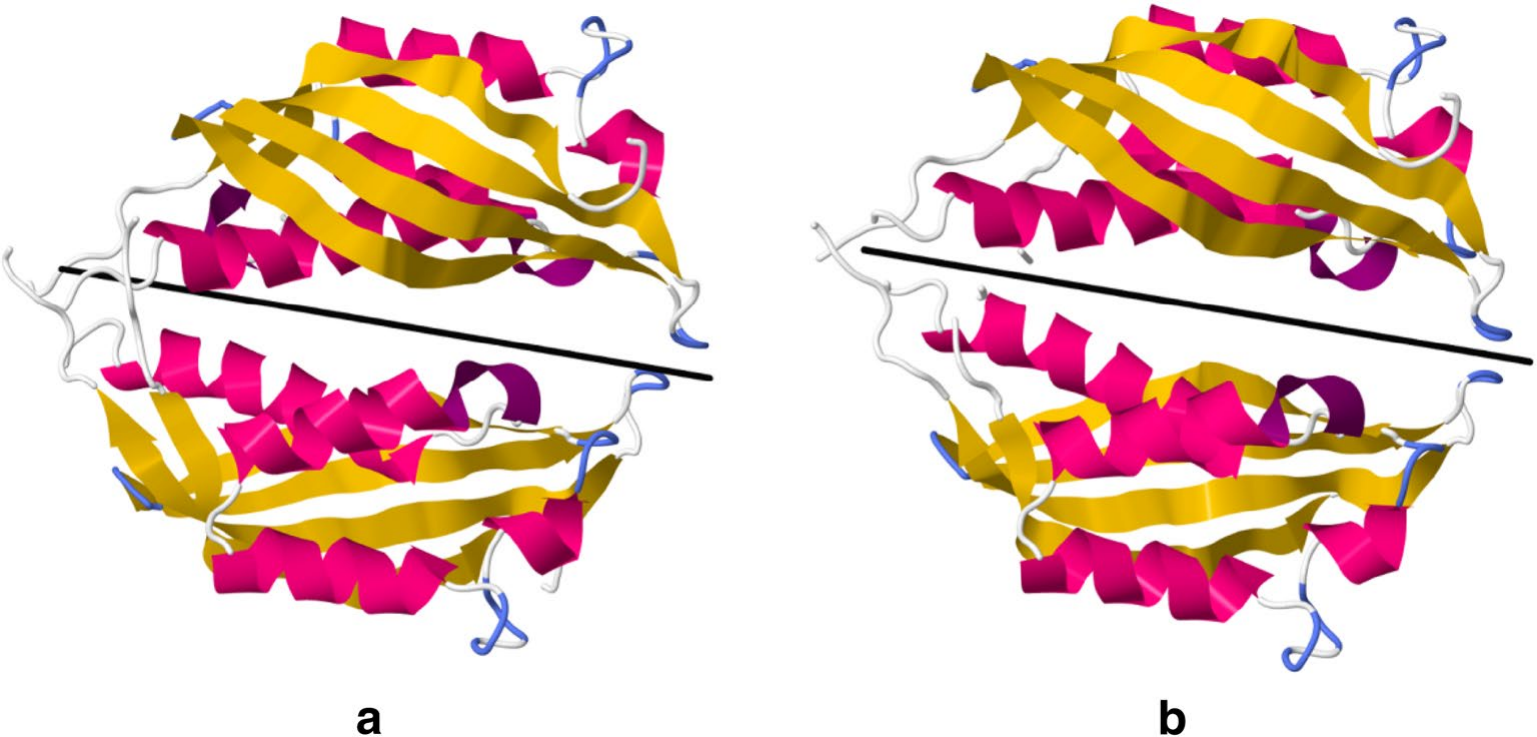
The homodimer (*R*. *typhi* RvhB8-II) (PDB-ID: 4O3V) is characterized with *S*(*C*_2_) = 1.1261. **a** Original structure and **b** nearest symmetric structure. The black line represents the direction of the symmetry axis for the nearest symmetric structure. See Additional file 1: Figure S2 for ball and sticks models of the structures

Figure 3 exemplifies the calculation for the crystal structure of the VirB8-like protein, *R*. *typhi* RvhB8-II homodimer (PDB ID: 4O3 V) [39]. The left structure is the original one, and the right structure is the nearest symmetric structure with *S*(*C*_2_) = 1.1261. While the differences may appear minor with the ribbons view, one should bare in mind that these are more significant at the atoms level (see a ball and sticks view in Additional file 1: Figure S2).

**Fig. 4:**
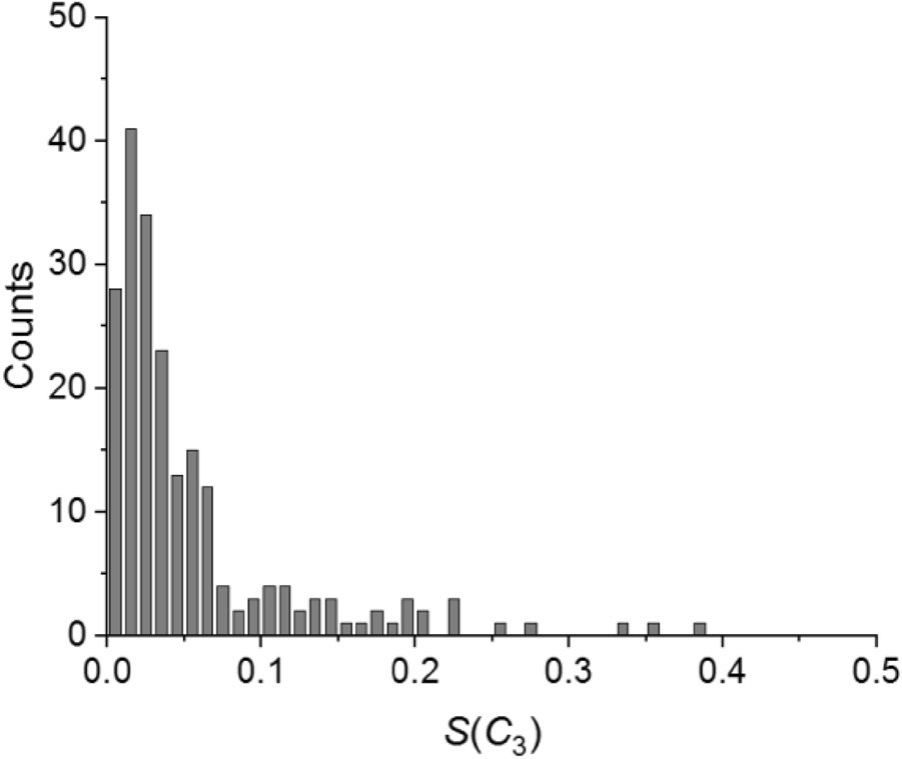
*S*(*C*_3_) distribution for the set of 214 protein trimers. Bin size was set to 0.01. The right tail of the distribution is not shown in order to increase the visibility

Returning to Table 1 we note that the standard deviation of each set is much higher than the corresponding mean value. This is resulting from the fact that the CSM is always positive and its distribution has a long tail as exemplified in Fig. 4 for our set of 214 trimers.

Another interesting point apparent from Table 1 is that for even homomers, distortion increases with the order of the rotational symmetry. For tetramers—the mean deviation from *C*_2_ symmetry (0.0442) is smaller than the mean deviation from *C*_4_ symmetry (0.1001). Likewise the mean distortion of the set of hexamers increases in the order: *S*(*C*_2_) <* S*(*C*_3_) <* S*(*C*_6_). The same trend is seen when looking at either the minimum, median or maximum CSM values. This is to be expected—for a hexamer to be perfectly *C*_6_-symmetric, all peptides must be symmetrically aligned around the rotation axis. That is, all peptides must attain a symmetrically equivalent conformation. However, to obtain a *C*_2_-symmetry, it is enough that three of the peptides will be symmetrically equivalent to the other three. That is, the structure has more degrees of freedom because the peptides at each triplet need not be perfectly symmetric with respect to each other.

### Finding the correct permutation

**Fig. 5:**
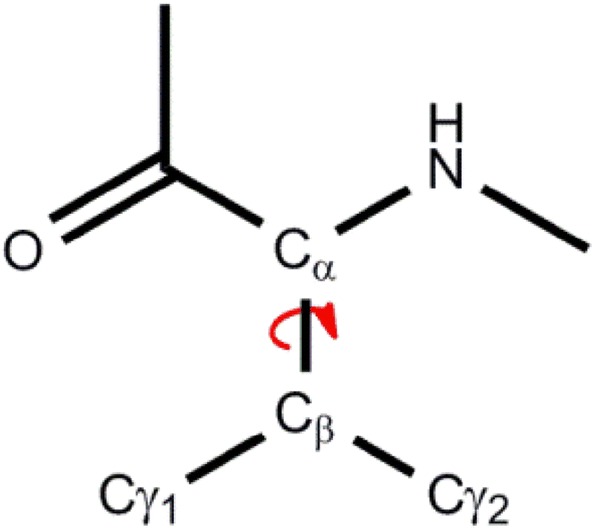
Val has two C_γ_ atoms. The free rotation around the C_α_–C_β_ bond, give rise to two possible permutations of these carbon atoms with those of another Val on an equivalent peptide

As we have described above, our algorithm guarantees that the atom’s permutation does not break the peptides: Each peptide is carried in its entirety to another peptide, and therefore we have two levels of permutations: The permutation of equivalence classes of atoms (Fig. 1), and the permutation of the peptides (Fig. 2). Let us look at these permutations more closely. For most of the atoms, their order in the sequence determines their permutation. That is, a C_α_ of Gly can only be interchanged with a C_α_ of Gly that has the same sequence number on the equivalent peptide. However, Val for example, has two C_γ_ atoms (see Fig. 5). These give rise to two possible permutations: either C_γ1_(A) → C_γ1_(B) or C_γ1_(A) → C_γ2_ (B) where A and B are the equivalent peptides. Other possibilities for such permutations are the ring carbons of Phe and Tyr, The C_δ_’s of Leu, the two nitrogen atoms at the tail of Arg and the two oxygen atoms at the tail of Asp and Glu. If hydrogen atoms exist in the PDB file, larger equivalence groups will result (e.g., for methyl groups at the edge of the side chains). The atoms permutation is kept as a separate list for each combination of two peptides. After the peptides permutation is found, the final total permutation is constructed by linking the peptides permutation with the relevant atoms permutations. In what follows we describe the possibilities for peptide permutations, and the differences between them.

Finding the permutation of the peptides follows Eq. (6) described above. For dimers and trimers, finding the permutation of the peptides is straightforward: A → B for dimers and A → B→C for trimers (note that A → C→B is an equivalent permutation). However as the number of peptides in a protein increases, it is not clear a priori which permutation will lead to a smaller CSM. A peptide permutation that follows the order of the peptides in the PDB file was found for 45% of the tetramers, 69% of the pentamers and 81% of the hexamers. In other words, relying on the PDB file order of peptides can lead to an error of up to 55% of the tetramers, 31% of the pentamers and 19% of the hexamers. Nevertheless, larger data sets may alter these numbers. Additional file 1: Tables S2–S4 present the specific permutations and their frequencies for our sets of tetramers, pentamers and hexamers

We continue by testing the differences between the best permutation of the peptides and atoms, to the sequence-ordered permutation of the atoms, in which the atoms are interchanged according to their serial numbers in the PDB file, and to the permutation found by the greedy algorithm. In both cases all possible permutations of the peptides were taken into account. These differences, although important, do not affect the permutation of the peptides. Starting with the sequence-ordered permutation of the atoms, we found that it generally leads to higher CSM values as compared with the ones found by the Hungarian algorithm. That is, it finds the protein to be less symmetric than it really is. Table 2 presents the results of these comparisons in terms of the relative error defined by:

7 $${\text{Relative}}\;{\text{Error}} = 100 \cdot \frac{{\left| {CSM_{full} - CSM_{{sequence{ - }ordered}} } \right|}}{{CSM_{full} }}$$

**Fig. 6:**
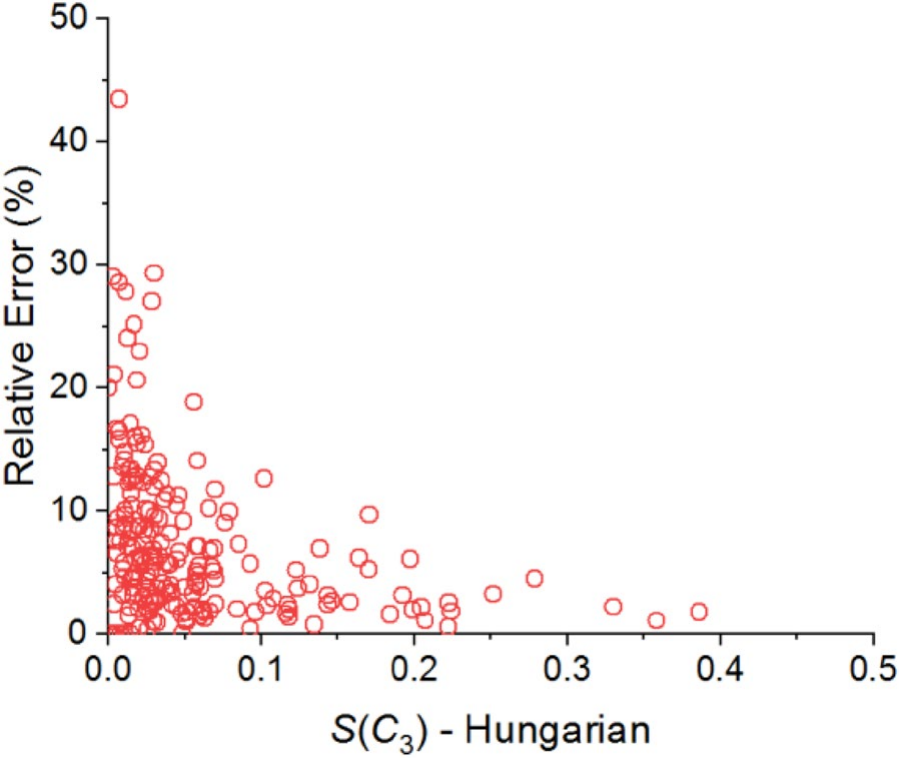
Relative error of *S*(*C*_3_) calculated for the dataset of 214 protein trimers resulting from using the sequence-ordered permutation as opposed to the permutation found by the Hungrian algorithm

As is evident, using the sequence-ordered permutation adds a median relative error of 4–10%, but the maximum error is much higher and can be as high as 49%. Two comments are in place here. First, the higher errors are more abundant when the measure itself is low, that is, the protein is highly symmetric. This is resulting from the definition of the relative error in Eq. (7). As an example, Fig. 6 presents the relative error as a function of *S*(*C*_3_) for the set of trimers. Second, in few cases the sequence-ordered permutation does provide the same CSM value as with the Hungarian algorithm. This was obtained for 11 out of 214 trimers (5%) and 1 out of 31 tetramers (3%) in the calculation of *S*(*C*_4_). Note that the minimum relative error of *S*(*C*_2_) for the same set of tetramers is not zero.

**Table 2:** Descriptive statistics of the relative deviation of the CSM resulting from the sequence-ordered permutation of the atoms as compared with the Hungarian algorithm

Set	CSM	N	Mean (%)	Standard deviation (%)	SE of mean (%)	Minimum (%)	Median (%)	Maximum (%)
Dimers^a^	*S*(*C*_2_)	193	6.7	6.7	0.5	0.1	4.5	37.3
Trimers	*S*(*C*_3_)	214	6.9	6.6	0.5	0.0	5.1	43.5
Tetramers	*S*(*C*_2_)	31	12.8	10.6	1.9	0.4	10.0	49.3
	*S*(*C*_4_)	31	9.9	10.1	1.8	0.0	6.6	42.4
Pentamers	*S*(*C*_5_)	51	5.7	5.1	0.7	0.1	4.6	21.5
Hexamers	S(C_2_)	16	9.0	6.9	1.7	2.4	6.6	25.0
	S(C_3_)	16	7.7	5.2	1.3	2.2	6.4	20.8
	*S*(*C*_6_)	16	7.1	4.9	1.2	1.9	6.1	21.0

^a^Statistical analysis was done on 193 out of 194 dimers. One dimer, with PDB-ID 2AJQ, is highly symmetric with *S*(*C*_2_) = 0.0001 for the Hungarian algorithm and 0.0004 for the sequence-ordered permutation, led to an error of 300%. It was therefore considered as an outlier and excluded from this calculation

Similarly to the analysis presented above, a comparison with the greedy algorithm was conducted. In all cases but one, the greedy algorithm found a higher CSM value as compared with the Hungarian algorithm, that is a permutation that leads to a less symmetric structure. Table 3 presents the comparison of the Hungarian algorithm and the greedy algorithm. Here the relative errors are lower, with a median error of 1% and up to 3% and a maximum error of 3% and up to 10%. This makes sense as the greedy algorithm does attempt to find a better permutation than the sequence-ordered permutation, although it does not succeed in all of the cases. A zero relative error has been obtained in higher percentages as compared with the sequence-ordered permutation: 9% of the dimers, 12% of the trimers, 26% for *S*(*C*_2_) of the tetramers and 19% for *S*(*C*_4_), 6% of the pentamers, none for *S*(*C*_2_) and *S*(*C*_3_) of the hexamers and 13% for *S*(*C*_6_) of the hexamers. Altogether, we can estimate that the greedy algorithm for finding the permutation of the atoms is equivalent to the Hungarian algorithm in up to 26% of the calculations.

**Table 3:** Descriptive statistics of the relative deviation of the CSM resulting from a greedy algorithm of the atoms as compared with the Hungarian algorithm

Set	CSM	N	Mean (%)	Standard deviation (%)	SE of mean (%)	Minimum (%)	Median (%)	Maximum (%)
Dimers	*S*(*C*_2_)	194	1.5	1.3	0.1	0.0	1.2	7.1
Trimers	*S*(*C*_3_)	214	1.5	1.1	0.1	0.0	1.3	5.6
Tetramers	*S*(*C*_2_)	31	2.0	1.9	0.3	0.0	1.7	6.8
	*S*(*C*_4_)	31	1.1	1.0	0.2	0.0	1.0	3.3
Pentamers	*S*(*C*_5_)	51	2.1	1.9	0.3	0.0	1.6	9.8
Hexamers	S(C_2_)	16	2.9	2.1	0.5	0.5	2.2	7.9
	S(C_3_)	16	1.6	1.2	0.3	0.3	1.3	3.9
	*S*(*C*_6_)	16	2.5	1.6	0.4	0.0	2.6	5.1

### Calculation time

**Fig. 7:**
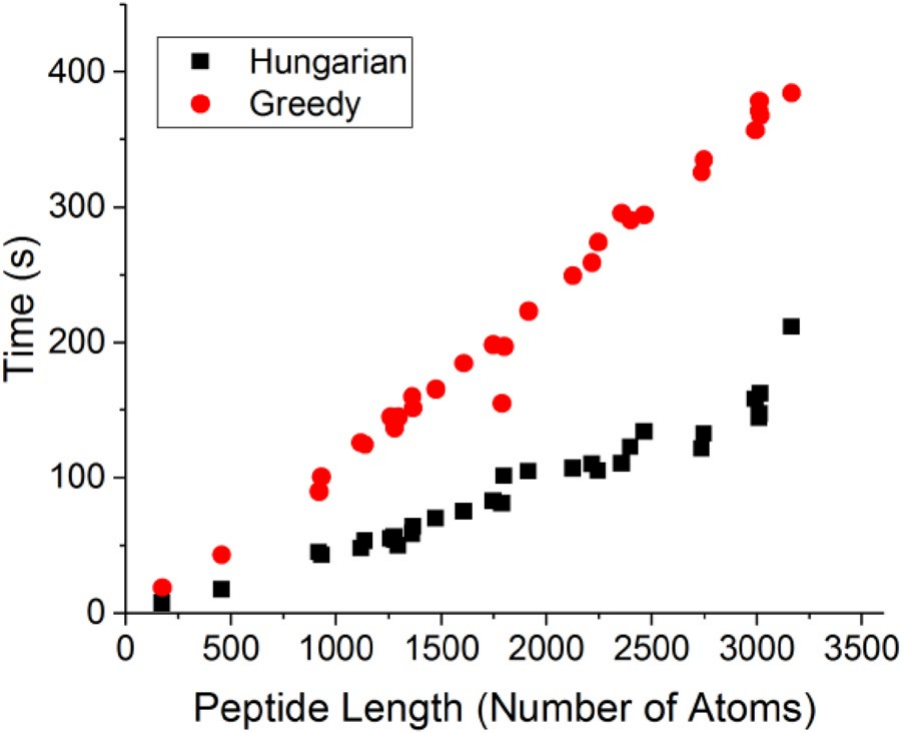
Calculation time of *S*(*C*_4_) for the tetramers dataset using the many-chains algorithm. Red circles: greedy algorithm. Black square: Hungarian algorithm

As an estimation for the speed of the calculation we present in Fig. 7 the real time for calculating *S*(*C*_4_) for our set of tetramers as a function of the number of atoms in each peptide. Generally, for short proteins, time increases linearly with size. As the number of atoms increases, the time dependency deviates from linearity. The time range was 9 s for the shortest protein with 174 atoms in each peptide, and up to ca. 5 min for a protein with 3166 atoms in each peptide. We note that the number of iterations the code performs in order to find the best permutation is typically small, between 2 and 4. All calculations were performed using one core of an 8-cores Linux machine with 64 GB RAM memory.

### CSM and RMSD

**Fig. 8:**
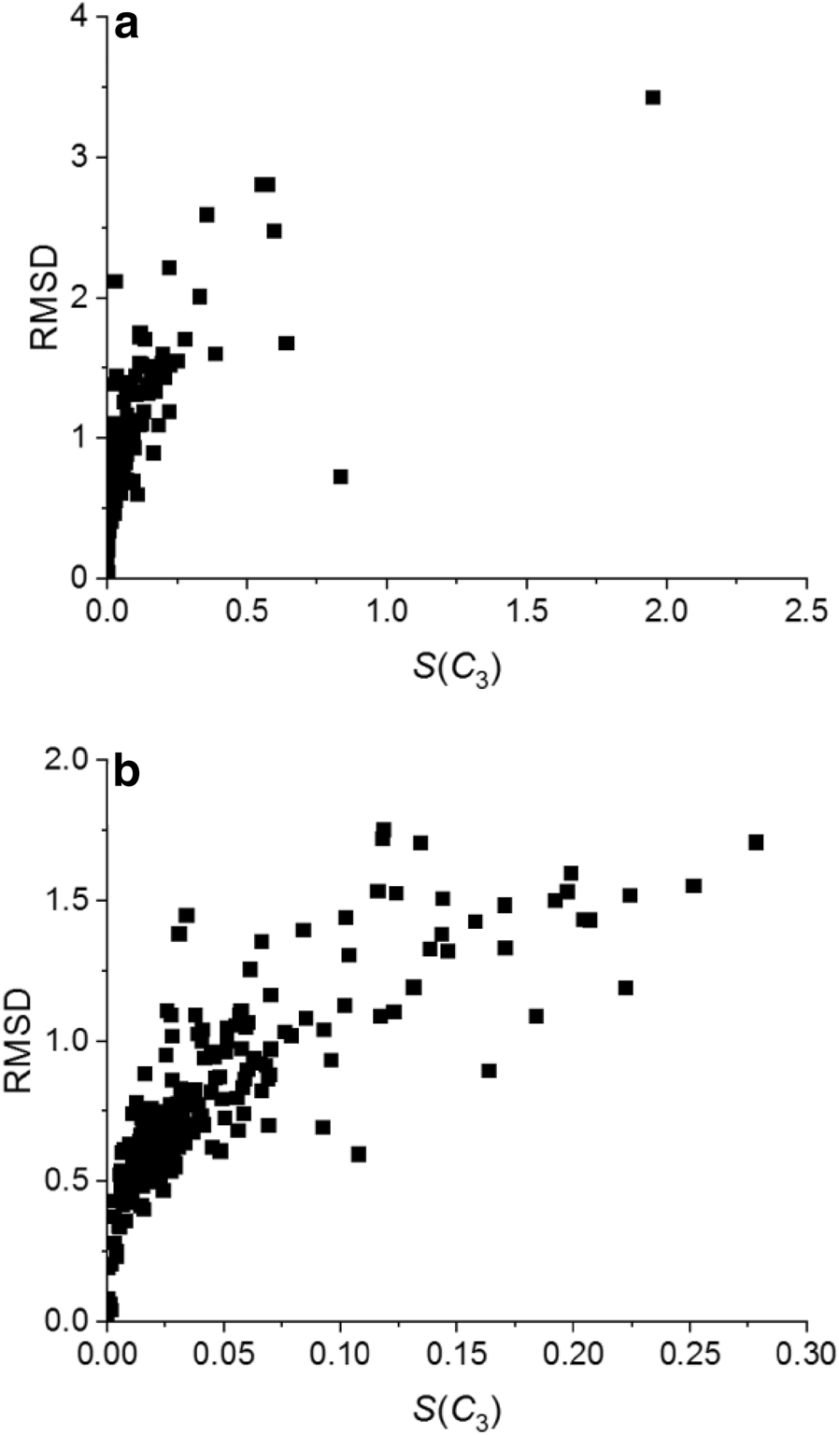
RMSD versus *S*(*C*_3_) for the set of trimers. **a** Full range and **b** inset

Symmetry breaking is commonly analyzed in the literature by superposing the protein subunits and assessing the root mean square deviation (RMSD) between them [26, 27]. The RMSD, like the CSM is zero for perfect symmetry and increases as the distortion increases. As seen in Fig. 8a for our set of trimers, both the RMSD (calculated with MOE [40] for all atoms) and the CSM increases with the deviation from *C*_3_ symmetry, but are not quantitatively correlated. For highly symmetric structures the correlation improves though it remains qualitative (Fig. 8b). Similar results were obtained for the other sets of homomers.

## Conclusions

Continuous symmetry and chirality measures determine the distortion level of a structure by searching for the nearest symmetric (or achiral) structure and calculating the distance between the two structures. The approximate algorithm presented here provides significant improvements over pervious codes in terms of accuracy, speed of the calculation, and the scope of molecular structure complexity it can handle. As has been shown here it can be used as a robust and versatile molecular descriptor of protein structure. Symmetry is an important advantageous for protein structure, yet not trivial to achieve. With an accurate and efficient tool to estimate this symmetry one opens the door to understand where and why nature fails to achieve perfect symmetry and what functions do imperfection serve. Applications of the methods include characterization of the three-dimensional structure of proteins in the solid state or in solution, analysis of conformational changes during dynamical processes and exploration of quantitative structure–activity relationships. Modification of the methods for a robust analysis of non-biological large molecules as well as nanomaterials and molecular clusters is currently in progress.

## Additional file

**Additional file 1.** Supplementary material.

## Data Availability

A list of PDB-IDs used in this study is given in the Additional file 1. An online CSM calculator is available at: http://csm.ouproj.org.il. Project name: proteincsm. Project home page: https://github.com/continuous-symmetry/proteincsm. Archived version: 1.0.1. Operating system(s): Linux, Windows. Programming language: Python, c++. Other requirements: unzipping package like bzip2, OpenBabel (requires X11 libraries), c++ compiler, conda, numpy. License: GNU-GPL version 2. Any restrictions to use by non-academics: Not applicable. Project name: pdb_prep. Project home page: https://sagivba.github.io/pdb_prep/. Archived version: 0.0.8.4. Operating system(s): Linux. Programming language: Python. Other requirements: click. License: BSD 2-Clause. Any restrictions to use by non-academics: Not applicable.

## Abbreviations

CSM: continuous symmetry measure
RMSD: root mean square deviation
