# Supplementary material for: Improved algorithms for quantifying the near symmetry of proteins: complete side chains analysis
Source: J Cheminform. 2019 Jun 6;11:39. doi: 10.1186/s13321-019-0360-9 (PMC6551912; doi:10.1186/s13321-019-0360-9)
Supplement: Supplementary file 1 — Additional file 1. Supplementary material. [file 13321_2019_360_MOESM1_ESM.docx]

**Supplementary Material**

Improved algorithms for quantifying the near symmetry of proteins: Complete side chains analysis

Inbal Tuvi-Arad^1^* and Gil Alon^2^*

^1^Department of Natural Sciences, The Open University of Israel, Raanana, 4353701, Israel.

^2^Department of Mathematics and Computer Sciences, The Open University of Israel, Raanana, 4353701, Israel.

| **Content** | **Page** |
| --- | --- |
| The assignment problem and the Hungarian algorithm | S2 |
| Figure S1: Inaccuracies resulting from the greedy algorithm - an example | S3 |
| Lists of PDB –IDs used in this study | S4 |
| Figure S2: Ball & sticks models of the homodimer with PDB-ID: 4O3V | S6 |
| Table S1: Peptides permutations for the set of 31 tetramers | S7 |
| Table S2: Peptides permutations for the set of 51 pentamers | S7 |
| Table S3: Peptides permutations for the set of 16 hexamers | S8 |
| References | S8 |

**The assignment problem and the Hungarian algorithm**

We summarize here, for the readers' convenience, the relevant information about the assignment problem and its effective solution.

The assignment problem is classically stated as follows: A manager has to assign $n$ workers with $n$ tasks (with $n$ being a positive integer). Each worker has a known efficiency for each of the tasks (given by a real number). How can the manager assign the tasks such that the sum of the efficiencies is maximal?

In more concrete terms, let us denote by $a_{ij}$ the efficiency of worker no. $i$ on task no. $j$. We combine these numbers to form a matrix $A=(a_{ij})$. Each assignment of the tasks corresponds to a choice of one entry in each row, such that in each column there is exactly one chosen entry. Such a choice is called a *generalized diagonal* of the matrix. We are looking for a generalized diagonal whose sum is maximal. Equivalently, we are looking for a permutation $\pi$ of $\{1,2,..,n\}$ such that the sum $\sum_{i=1}^{n} a_{i\pi(i)}$ is maximal (in this representation, $\pi(i)$ is the location of the chosen entry at row no. $i$). As there are $n!$ such permutations, and this number grows very quickly with $n$, we look for a more efficient algorithm for finding the optimal permutation.

Such an algorithm is the Hungarian algorithm,[1] whose running time has order of magnitude $n^{3}$. Let us describe it briefly. The problem may be easily transformed to the problem of finding a generalized diagonal with *minimal* sum, for a matrix whose entries are nonnegative integers.

The algorithm finds such a permutation using the following crucial observation: If one adds a constant real number (either positive or negative) to each entry of a certain row, or a certain column of the matrix, the optimal permutation does not change. The Hungarian algorithm applies such changes over and over (by a strategy that we shall not describe here) until the matrix has a generalized diagonal whose entries are all zeros (and still has nonnegative entries everywhere). For such a matrix, it is evident that the generalized diagonal with the zeros is the optimal one.

**Inaccuracies resulting from the greedy algorithm – example with two atoms**

If an equivalence class has two atoms, $Q_{1}$ and $Q_{2}$, which are positioned, along with their symmetric images $TQ_{1},TQ_{2}$ as in Figure S1a, then the greedy algorithm would assign $\pi(2)=2$, and consequently $\pi(1)=1$. The contribution of this choice to the sum in Eq. (4) is $|Q_{1}-TQ_{1}|^{2}+|Q_{2}-TQ_{2}|^{2}$, whereas the contribution of the opposite choice $\pi(1)=2, \pi(2)=1$(see Figure S1b), is $|Q_{2}-TQ_{1}|^{2}+|Q_{1}-TQ_{2}|^{2}$. In this particular configuration we have $|Q_{1}-TQ_{1}|^{2}+|Q_{2}-TQ_{2}|^{2}>|Q_{2}-TQ_{1}|^{2}+|Q_{1}-TQ_{2}|^{2}$, so the choice of the greedy algorithm does not minimize Eq. (4).

**Figure S1.** Two atoms (Q_1_ and Q_2_) and their symmetric images (TQ_1_ and TQ_2_). Arrows represent the choice of permutation with: **a.** The greedy algorithm; **b.** The Hungarian algorithm.

**List of PDB –IDs used in this study**

Homodimers

1AJ8, 1CKU, 1DXG, 1E5X, 1EGI, 1EK6, 1EX0, 1EZG, 1F75, 1I07, 1JK6, 1LQ9, 1O1H, 1O69, 1OAS, 1OKI, 1QF8, 1SR7, 1T33, 1UCR, 1VFR, 1W2I, 1WKQ, 1WLS, 1WMX, 1WTJ, 1Y44, 1Y6H, 1YB1, 2A4K, 2AL1, 2BDW, 2C2X, 2CAR, 2CWK, 2DDM, 2E0A, 2EL7, 2F5G, 2FA5, 2FE7, 2FYX, 2G9Z, 2GPC, 2HIN, 2ISW, 2IU4, 2J8W, 2JAQ, 2NP3, 2NVM, 2NYI, 2OD0, 2P0U, 2PLR, 2PYW, 2Q20, 2QSI, 2VG0, 2XR4, 2YG3, 2ZQ0, 3CT9, 3DM7, 3DMC, 3FMB, 3GZR, 3H3H, 3HAM, 3HHF, 3I9L, 3IGF, 3IHM, 3ILX, 3IN6, 3JSW, 3KDG, 3KH8, 3KUV, 3L46, 3L7O, 3LAC, 3LCR, 3LGJ, 3LMB, 3LNT, 3LQS, 3M1U, 3MUQ, 3MXJ, 3MZ2, 3N9S, 3NRR, 3O5U, 3O8D, 3OI8, 3OJI, 3OYT, 3P2C, 3PM7, 3QW3, 3R12, 3R1Z, 3SY6, 3T3A, 3T4E, 3TR3, 3UNC, 3V4C, 3VGJ, 3VP6, 3VV1, 3WAN, 3WEU, 3WRB, 3WXB, 3ZYY, 4A01, 4AG7, 4AW3, 4B7V, 4C4N, 4DBH, 4DO4, 4EBG, 4EGS, 4EP4, 4FU0, 4GS1, 4GYM, 4H30, 4H3Z, 4H51, 4HEH, 4HWV, 4I4O, 4INE, 4JLE, 4L9I, 4LQC, 4M9D, 4NDK, 4O0C, 4O3V, 4OI3, 4P04, 4Q9B, 4QGR, 4QRI, 4R82, 4RPA, 4S26, 4TR6, 4U0S, 4U13, 4UUU, 4W5K, 4XUK, 4Y86, 4Z39, 4Z9H, 4ZPZ, 4ZQS, 4ZWO, 5AEC, 5AWI, 5B0H, 5C77, 5C8Z, 5E3J, 5E5C, 5ER9, 5FAS, 5GJO, 5GMH, 5GRM, 5H78, 5H9S, 5HQW, 5HSX, 5I4Z, 5IPY, 5J0K, 5JG7, 5JIP, 5JOD, 5K3X, 5L2R, 5V7I, 5VAA, 5WBM, 5X7Q, 5XKO, 6AWA.

Homotrimers

1A12, 1A88, 1C5E, 1DUV, 1EL6, 1H7Z, 1HX6, 1JLJ, 1KHT, 1KNC, 1KRR, 1LUA, 1O91, 1OCX, 1PM4, 1PPR, 1Q5H, 1QBZ, 1QD9, 1TCV, 1U2M, 1V4N, 1V8D, 1VMK, 1XHO, 1XRG, 1YF9, 1YU4, 1ZRU, 2A5Z, 2BCM, 2BHW, 2BRJ, 2BT9, 2C0A, 2CHC, 2CZ4, 2D39, 2E2A, 2EKM, 2F0C, 2F4N, 2FB5, 2FEF, 2GTR, 2I6U, 2I9D, 2IDX, 2IEX, 2IG8, 2IGT, 2INU, 2IS8, 2IUM, 2O4V, 2O66, 2OTM, 2P2L, 2P4S, 2P90, 2Q01, 2Q0T, 2RGQ, 2RIE, 2UZH, 2VES, 2WN3, 2WR1, 2WW2, 2X3H, 2XC1, 2XGF, 2XU8, 2YNY, 2YZJ, 2ZBV, 2ZHZ, 3C6V, 3CJ8, 3CPX, 3CQO, 3DA0, 3EMF, 3EXW, 3F4F, 3FOB, 3FRO, 3FUC, 3FUY, 3G64, 3GKB, 3GKE, 3H35, 3H6X, 3HJJ, 3HTN, 3IRS, 3JQY, 3KE4, 3KTO, 3LAO, 3LGI, 3MAE, 3NKE, 3NTN, 3OL0, 3PZK, 3R1W, 3R7F, 3R9T, 3RWN, 3SWF, 3SWX, 3T71, 3TK8, 3TY1, 3VBJ, 3VCZ, 3W92, 3WV7, 3ZJB, 3ZXP, 4A0T, 4B0H, 4CQJ, 4DI1, 4DZN, 4E38, 4EZC, 4F23, 4FAY, 4FIO, 4FN7, 4GOM, 4GVQ, 4H31, 4H41, 4HI1, 4HIZ, 4HUR, 4HZE, 4I6V, 4IWG, 4JBZ, 4JCU, 4JDN, 4K3W, 4LIY, 4MC5, 4MEJ, 4MT4, 4N72, 4NF2, 4NNQ, 4NSN, 4OGG, 4OIF, 4OJ5, 4OOP, 4PBP, 4Q1K, 4R9J, 4RHM, 4RU5, 4RWW, 4TME, 4U19, 4U5R, 4UE0, 4UOF, 4UOZ, 4USI, 4W1T, 4WOL, 4XL8, 4Y65, 4YSE, 4Z0M, 4ZU2, 5AZP, 5B2E, 5B8F, 5CMU, 5CUM, 5CXD, 5EIL, 5F7X, 5F9K, 5FUS, 5G47, 5GYL, 5HT7, 5JBX, 5JQM, 5JS4, 5K21, 5L12, 5M9F, 5MAB, 5N2C, 5O65, 5ONU, 5SUV, 5T9Y, 5TB7, 5V0Z, 5V13, 5VBX, 5XEA, 5XL8, 5Y5Q, 6B1K, 6EGU, 6EYU.

Homotetramers

1J8D, 1NC7, 2BWG, 2GIX, 2H16, 2V9N, 2YWM, 2ZRU, 3BPZ, 3D9S, 3E8M, 3I6B, 3IJ5, 3LBW, 3PVS, 3RQA, 3RXZ, 3TIA, 3VR0, 4AVF, 4B7R, 4BHV, 4H53, 4HGN, 4IXH, 4KOP, 4LY4, 4M6R, 4RJE, 5FGS, 5HUM.

Homopentamers

1EJB, 1K5J, 1KZ1, 1T0T, 1VDH, 1Y2I, 2BJ0, 2C92, 2F59, 2RCF, 2RLD, 2WN9, 3CK6, 3DRZ, 3DWA, 3KCU, 3KLY, 3NN1, 3P05, 3TDS, 3WTO, 3WZ2, 4AFH, 4AVS, 4DMI, 4HFI, 4J07, 4J4V, 4JW0, 4MJ0, 4MOZ, 4N8F, 4N8M, 4PCG, 4PCH, 4PND, 4RHS, 4U60, 4UOT, 4WD8, 5A12, 5AFN, 5CPU, 5HLR, 5INR, 5JBL, 5JIE, 5L37, 5LOQ, 5WHT, 5Y9E.

Homohexamers

1E9R, 1SVM, 2A10, 2EKD, 2GJV, 2XF7, 3INZ, 3KVP, 3SSS, 3WU4, 4BLP, 4KVT, 4OX7, 4PN9, 4TLA, 4X9C.


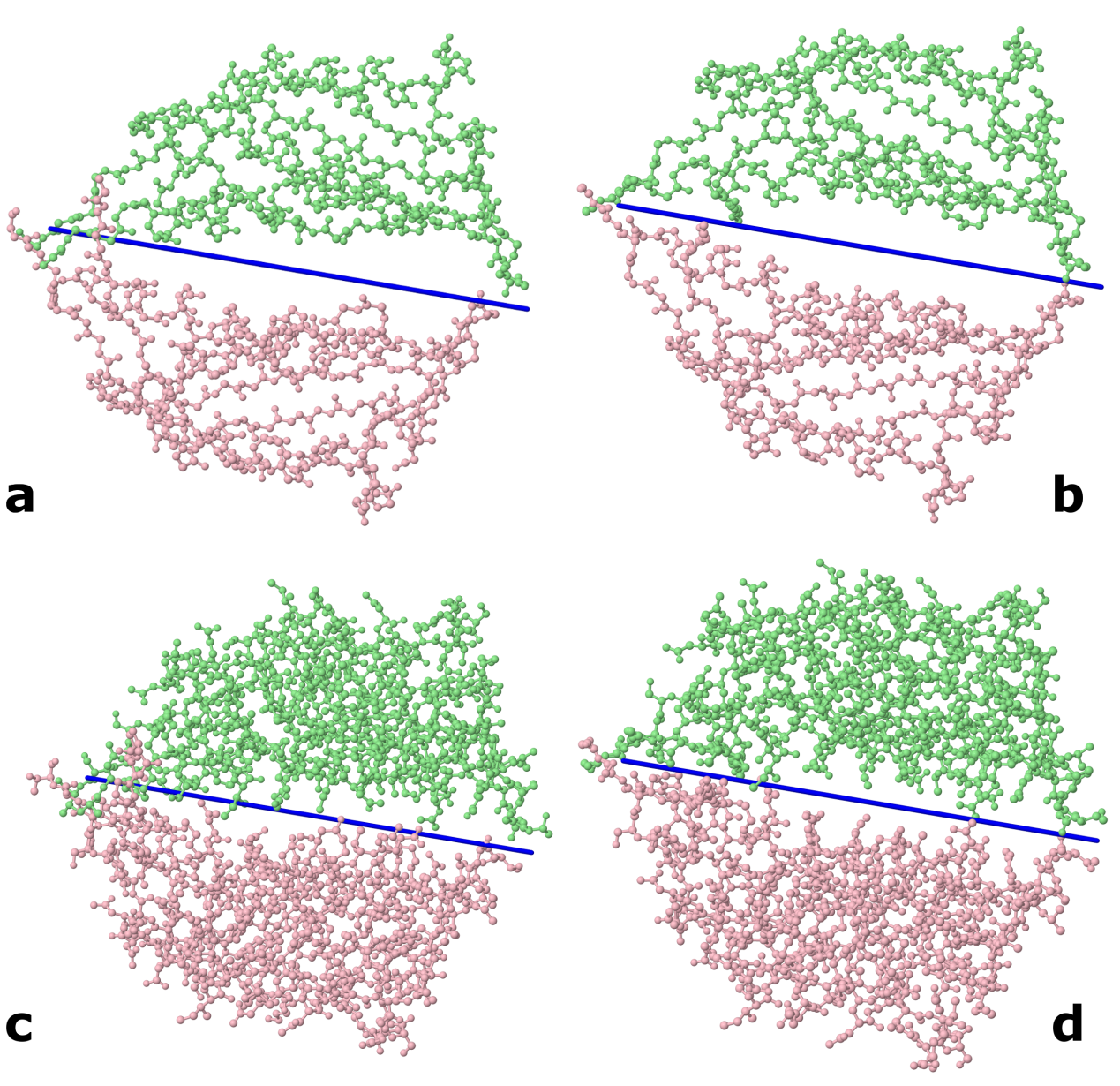


**Figure S2**. Ball & sticks models of the homodimer with PDB-ID: 4o3v (*R*. *typhi* RvhB8-II) is characterized with *S*(*C*_2_) = 1.1261. **a.** Backbone of the original structure. **b.**: Backbone of the nearest structure with *C*_2_ symmetry. **c.** Original structure – all atoms. **d.** Nearest symmetric structure – all atoms. The blue line represents the direction of the symmetry axis of the nearest symmetric structure.

**Peptide Permutations Tables**

**Table S1.** Peptides permutations for the set of 31 tetramers.

| **Permutation** | | **Frequency** |
| --- | --- | --- |
|  | A→B→C→D | 14 |
|  | A→B→D→C | 9 |
|  | A→C→B→D | 8 |

**Table S2**. Peptides permutations for the set of 51 pentamers.

| **Permutation** | | **Frequency** |
| --- | --- | --- |
|  | A→B→C→D→E | 35 |
|  | A→B→D→C→E | 3 |
|  | A→B→E→D→C | 5 |
|  | A→C→B→D→E | 1 |
|  | A→C→D→B→E | 3 |
|  | A→D→C→E→B | 3 |
|  | A→D→B→C→E | 1 |

**Table S3**. Peptides permutations for the set of 16 hexamers.

| **Permutation** | | **Frequency** |
| --- | --- | --- |
|  | A→B→C→D→E→F | 13 |
|  | A→B→D→E→F→C | 1 |
|  | A→C→D→E→B→F | 1 |
|  | A→D→C→E→B→F | 1 |

**References**

1. Munkres J (1957) Algorithms for the Assignment and Transportation Problems. Journal of the Society for Industrial and Applied Mathematics 5: 32-38.
